# Analysis of cardiovascular risk and carotid intima-media thickness in patients with psoriasis^☆^^☆☆^

**DOI:** 10.1016/j.abd.2019.07.004

**Published:** 2020-01-31

**Authors:** Elaine Cristina Faria Abrahão-Machado, José Alexandre Mendonça, Ana Carolina Belini Bazán Arruda, Luciana Bertoldi Nucci, Marcel Alex Soares dos Santos

**Affiliations:** a Dermatology Outpatient Clinic, Hospital da Pontifícia Universidade Católica de Campinas, Campinas, SP, Brazil; b Rheumatology Outpatient Clinic, Pontifícia Universidade Católica de Campinas, Campinas, SP, Brazil; c Psoriasis Outpatient Clinic, Hospital da Pontifícia Universidade Católica de Campinas, Campinas, SP, Brazil; d Postgraduate Program, Pontifícia Universidade Católica de Campinas, Campinas, SP, Brazil; e Discipline of Dermatology, Faculdade de Medicina São Leopoldo Mandic, Campinas, SP, Brazil

**Keywords:** Cardiovascular diseases, Carotid intima-media thickness, Methotrexate, Molecular targeted therapy, Psoriasis, Ultrasonography

## Abstract

**Background:**

Psoriasis is associated with atherosclerosis and increased cardiovascular risk. Currently, an automated ultrasound, called quantitative intima media thickness, has proven to be a useful method to evaluate subclinical atherosclerosis.

**Objectives:**

To compare increased cardiovascular risk in psoriasis patients receiving two types of treatments: Methotrexate and tumor necrosis factor inhibitor and to evaluate the correlation between the Framingham score and quantitative intima media thickness.

**Methods:**

Fifty patients with plaque psoriasis were selected from June 2017 to July 2018, divided into two groups, receiving methotrexate and tumor necrosis factor inhibitor. Measurement of abdominal circumference, blood pressure, body mass index and presence of metabolic syndrome were performed. Afterwards, the patients were evaluated for increased cardiovascular risk with the Framingham score and for the quantitative intima media thickness of the carotid arteries.

**Results:**

The mean age was 54.8 (±12.5) with a slight male predominance (58%). Overall, 84% of the patients had elevated waist circumference, 82% had a body mass index above ideal, and 50% had a metabolic syndrome. For the correlation between quantitative intima media thickness and Framingham Score, Pearson's linear correlation coefficient was 0.617 (*p* < 0.001), indicating a moderate to strong positive association.

**Study limitations:**

The protective effect of the therapies cited in relation to the increased cardiovascular risk was not evaluated.

**Conclusions:**

A moderate to strong positive association was found correlating the Framingham Score values with the quantitative intima media thickness measurement and it is not possible to state which drug has the highest increased cardiovascular risk.

## Introduction

Psoriasis (Ps) is a chronic and recurrent inflammatory disease associated with a wide range of systemic manifestations. It affects approximately 2–3% of the population.^1^ Systemic drugs, including methotrexate, acitretin, cyclosporine and immunobiological drugs, are used alone or in combination with other treatment modalities. The choice of the most appropriate therapy for each case involves an analysis of the severity of the disease together with the presence or absence of comorbidities.^2^

Systematic reviews evidenced the association of Ps with increased prevalence and incidence of Metabolic Syndrome (MS), as well as its individual components: obesity, dyslipidemia, Type 2 Diabetes Mellitus (DM2), and Systemic Arterial Hypertension (SAH) and increased Cardiovascular Risk (CVR).^3^ They also demonstrated that patients with severe Ps compared to those with the mild form of the disease presented higher chances for the development of MS. Therefore, PS has become an independent risk factor for cardiovascular events.^4^

The Framingham score is the most widely used theoretical framework that demonstrates a causal relationship with Cardiovascular Disease (CVD) and justifies the adequate stratification of CVR for the future occurrence of CVD.^5^ Carotid Ultrasound (US) can be used as one of the main methods of noninvasive evaluation in the diagnosis of atherosclerotic carotid disease. It is characterized by being a good, dynamic, radiation-free and low-cost method.^6^

The use of the same for calculating carotid intima-media thickness by an automated ultrasound called Quantitative Intima Media Thickness (QIMT) is an important parameter to identify subclinical atherosclerotic disease in patients with or without risk factors. Differently from the other carotid artery thickness assessment techniques, this method is not examiner-dependent because the result is given by this software and not measured manually.^7^

In Ps, the inflammatory events that are perpetuated in the bloodstream probably influence the onset of lesions in the vascular endothelium and the development of atherosclerosis. From this same theory, the hypothesis arises that, by decreasing the inflammatory load, it would consequently decrease the risk of the onset of CVD.^8^ The concept of Ps as a systemic disease directed the scientific community to investigate the influence of therapies for Ps in the evolution of these more prevalent comorbidities.

The purpose of this study is to compare CVR in psoriasis patients receiving two types of treatments: methotrexate (MTX) and tumor necrosis factor inhibitor (TNF-i) and to evaluate the correlation between the Framingham score and QIMT.

## Methods

This study was a cross-sectional observational study, carried out from June 2017 to July 2018 in PUC-Campinas, with 50 patients with moderate to severe plaque psoriasis Psoriasis Area Severity Index (PASI) ≥ 10 and aged 20 years or over were divided into two groups:

Group 1: 25 patients receiving MTX for more than 6 months;

Group 2: 25 patients receiving TNF-i (infliximab or adalimumab) for more than 6 months.

We evaluated the measurement of abdominal circumference, Blood Pressure (BP), Body Mass Index (BMI) and presence of MS. Afterwards; they were evaluated for cardiovascular risk with the Framingham score and the QIMT of the two common carotid arteries.

Several groups have developed criteria for the diagnosis of MS, but the National Cholesterol Education Program (NCEP-ATP III) definition is the most widely used and recommended by the Brazilian Guideline on Diagnosis and Treatment of MS. Through a clinical and laboratory investigation, it aims to confirm the diagnosis of MS and to identify associated cardiovascular risk factors. Therefore, a combination of three of the following five parameters is required: triglyceride levels ≥ 150 mg/dL; HDL < 40 mg/dL for men and < 50 mg/dL for women; waist circumference > 88 cm for women and > 102 cm for men; fasting blood glucose ≥ 100 mg/dL and high blood pressure (BP ≥ 130 × 85 mmHg).

The Framingham score is calculated using information on age, LDL and HDL cholesterol, blood pressure, diabetes mellitus, and smoking for men and women. From the sum of the points of each factor is estimated the CVR in 10 years. Carotid ultrasound was performed using the gray scale or B-mode (SG), on the EsaoteMylab 50 machine, with a high resolution linear probe and frequency ranging from 3.5 to 10 MHz. The angulation to calculate the common carotid of the artery was less than 60°. Carotid Intima-Media Thickness (IMT) was determined using automated software that evaluates IMT by radiofrequency (QIMT).^9^ To obtain the QIMT, specific software was used to perform the semi-automatic reading and calculation of the expected relationship for each patient.

The image was obtained in the common carotid artery, bilateral, 10 mm proximally to the carotid bulb and in the final diastolic phase. The evaluation was performed with the measurement of the arterial thickening.

All examinations were performed by the same doctor with 10 years of experience in ultrasonography. The patient was in the supine position ≤ 45° to the contralateral side of the carotid artery under study and both carotid and transverse scans were evaluated.

All patients read and signed the Free and Informed Consent Form, approved by the Research Ethics Committee (voucher number: 061354/2017 and opinion number: 2,209,814) of the hospital involved.

### Statistical analysis

Descriptive and analytical statistical analyzes were carried out according to the proposed objectives. All analyzes were performed using SPSS Statistics software, version 17.0.

For the quantitative variables it was initially evaluated if they presented Normal distribution through descriptive analysis and the normality test of Shapiro–Wilk. The patient's age variable was described by the mean (± standard deviation), as it presented approximately Normal distribution and the time of drug use and the PASI values, with asymmetric distributions, were described by the median (interquartile range). Categorical variables were described by absolute (*n*) and relative (%) frequencies.

In the comparison analysis between the groups, Student's *t*-tests for the variable age of the patient, the Mann–Whitney test for the time of drug use and the PASI values were performed. For the other categorically described variables, Chi-Square tests or the Fisher's exact test were used.

For the evaluation of the CVR according to the treatment and the association between the CVR classifications, univariate and multivariable logistic regression analyzes were performed. The crude and adjusted odds ratios (OR – Odds Ratio) were calculated and their respective 95% Confidence Intervals (95% CI).

The concordance between the CVR measured by the Framingham score and the QIMT categorically was assessed by the Kappa coefficient and 95% CI. The calculations of sensitivity, specificity and accuracy were also performed. In addition, we assessed the association of CVR measured continuously by the Framingham score and the QIMT values through simple and multiple linear regression analysis. The level of significance (a) adopted was 5%, being considered statistically significant values of *p* < 0.05.

## Results

Fifty patients with moderate to severe Ps were evaluated, 50% were users of MTX (Group 1) and 50% of TNF-i (Group 2). The characteristics of the studied population are summarized in table 1. The majority of the patients were male (58%), white (88%) and mean age 54.8 (±12.5) years. The time of use of the medications was described through the median (1st–3rd quartiles) with a result of 36 (12–60) months.

**Table 1:** Characteristics of patients with psoriasis, total sample and according to the treatment performed.

Characteristics^a^	Total (*n* = 50)	MTX (*n* = 25)	TNF-i (*n* = 25)	*p*-Value
*Age (years)*	54.8 (±12.5)	59.4 (±10.7)	50.1 (±12.6)	0.007

*Gender*				0.252
Male	29 (58.0%)	12 (48.0%)	17 (68.0%)	
Female	21 (42.0%)	13 (52.0%)	8 (32.0%)	

*Skin color*				0.189
White	44 (88.0%)	24 (96.0%)	20 (80.0%)	
Brown	1 (2.0%)	0 (0.0%)	1(4.0%)	
Black	5 (10.0%)	1 (4.0%)	4 (16.0%)	

*Time of use of the drug (months)*	36 (12–60)	24 (12–60)	48 (21–84)	0.094

*Nutritional status*				0.564
Eutrophic	9 (18.0%)	4 (16.0%)	5 (20.0%)	
Overweight	26 (52.0%)	15 (60.0%)	11 (44.0%)	
Obese	15 (30.0%)	6 (24.0%)	9 (36.0%)	
PASI	12 (10–18)	10 (10–12)	16 (15–20)	<0.001
Mild (=10)	20 (40.0%)	15 (60.0%)	5 (20.0%)	
Severe (>10)	30 (60.0%)	10 (40.0%)	20 (80.0%)	0.004

a Data are presented as mean (± standard deviation), absolute frequency (%) or median (1st–3rd quartile). PASI, Psoriasis Area Severity Index; MTX, Methotrexate; TNF-i, infliximab and adalimumab.

The mean age of patients in the MTX group was 59.4 (±10.7) years and the TNF-i group was 50.1 (±12.6) years. The median time to use of the drug ranged from 24 (12–60) months for MTX users to 48 (21–84) months for the TNF-i group. Through BMI, worrying results were shown, since 82% of the patients in the study were overweight. Only 16% of Group 1 and 20% of Group 2 had the ideal weight for height. The PASI index at the start of treatment was greater than 10% for 60% of the patients, and in the MTX group this percentage was 40% and in the TNF-i group was 80%. The overall mean was 14.7 (±5.7), with 11.8 (±3.0) in Group 1 and 17.5 (±6.8) in Group 2. Due to the non-normality of the data, the Comparison of PASI between groups was performed using a non-parametric test, describing the medians (1st–3rd quartiles), indicating a statistically significant difference (*p* < 0.001) (Table 1).

Table 2 shows the comorbidities of these patients with Ps. Almost 40% of the patients had diagnosed SAH present in 48% of Group 1 and 28% of Group 2. DM2 in treatment was observed in 16% of patients, with 20% of MTX patients and 12% of TNF-i. Sixteen percent of study participants smoked, with the same value within the groups. Total cholesterol, HDL and LDL were altered in 50%, 76% and 30% of the patients, respectively, with no statistically significant difference between the groups. Values of triglycerides greater than or equal to ≥150 mg/dL were observed in 42% of the patients. Regarding previous cardiovascular disease, 14% of the patients had already presented a picture. Overall, 84% of the patients presented high waist circumference and 82% were overweight in relation to height, with 30% already considered obese, with no statistically significant difference between groups. We also analyzed the presence or absence of MS in these patients with Ps and in 50% of them it was present, with a higher percentage of MTX users (68%) than those receiving TNF-i (32%).

**Table 2:** Prevalence of comorbidities of patients with psoriasis, according to the treatment.

Comorbidities	Total *n* (%)	MTX *n* (%)	TNF-i *n* (%)	*p*-Value
Hypertension	19 (38.0%)	12 (48.0%)	7 (28.0%)	0.145^a^
Diabetes	8 (16.0%)	5 (20.0%)	3 (12.0%)	0.702^b^
Smoking	8 (16.0%)	4 (16.0%)	4 (16.0%)	1.000^b^
Total cholesterol ≥ 200 mg/dL	25 (50.0%)	13 (52.0%)	12 (48.0%)	0.777^a^
HDL ≤ 60 mg/dL	38 (76.0%)	17 (68.0%)	21 (84.0%)	0.185^a^
LDL ≥ 130 mg/dL	15 (30.0%)	7 (28.0%)	8 (32.0%)	0.758^a^
Triglycerides ≥ 150 mg/dL	21 (42.0%)	10 (40.0%)	11 (44.0%)	0.774^a^
Cardiovascular disease	7 (14.0%)	6 (24.0%)	1 (4.0%)	0.098^b^
High abdominal circumference	42 (84.0%)	23 (92.0%)	19 (76.0%)	0.247^b^
Overweight + Obesity	41 (82.0%)	21 (84.0%)	20 (80.0%)	1.000^b^
Obesity	15 (30.0%)	6 (24.0%)	9 (36.0%)	0.355^a^
Metabolic syndrome	25 (50.0%)	17 (68.0%)	8 (32.0%)	0.011^a^

a Chi-square test.

b Fisher's exact test.

Considering the prevalence of CVR according to the Framingham score, almost a quarter of the patients presented a high risk (odds of greater than 20% presenting coronary disease in 10 years), with 28% of Group 1 and 20% of Group 2; and more than 60% presented low risk, with a probability of less than 10% of developing a cardiovascular event in 10 years, with 56% in the MTX group and 72% in the biological group (Table 3).

**Table 3:** Prevalence of cardiovascular risk according to the Framingham score, carotid radiofrequency ultrasound (QIMT) and presence of carotid plaques.

	Total *n* (%)	MTX *n* (%)	TNF-i *n* (%)	*p*-Value
*Framingham score*				0.533^b^
Low	32 (64.0%)	14 (56.0%)	18 (72.0%)	
Intermediate	6 (12.0%)	4 (16.0%)	2 (8.0%)	
High	12 (24.0%)	7 (28.0%)	5 (20.0%)	

*QIMT*				0.239^a^
Normal	18 (36.0%)	11 (44.0%)	7 (28.0%)	
Changed	32 (64.0%)	14 (56.0%)	18 (72.0%)	

*Presence of carotid plates*				0.508^a^
Yes	12 (24.0%)	7 (28.0%)	5 (20.0%)	
No	38 (76.0%)	18 (72.0%)	20 (80.0%)	

a Chi-square test.

b Fisher's exact test.

Analyzing the carotid ultrasound, the result was worrisome because 64% of the patients presented QIMT above the upper limit of the age-adjusted values, signaling subclinical cardiovascular disease. In Group 1, 56% of the patients had thickening of the carotid intimal-medial layer and 28% in the carotid plaques. In Group 2, 72% of patients had altered QIMT results and 20% had carotid plaques. However, no association was found between CVR, calculated using the Framingham score and automated US and studied drugs, in other words, no statistically significant difference in risk between the two groups (Table 3).

Since the QIMT is still poorly explored in patients with Ps as a possible screening test for CVR assessment, we chose to evaluate the values found in this index continuously, correlating with the values of the Framingham score. The linear correlation coefficient of Pearson (*r*) was 0.617 (*p* < 0.001), indicating a positive association of moderate to strong. Table 4 describes the coefficients of the multivariate linear regression model.

**Table 4:** Association of the Framingham score vs QIMT.

Variables	Adjusted (95% CI)^a^	*p*-Value^b^
QIMT	8.77 (0.15, 17.39)	0.046
Age (years)	0.27 (0.17, 0.367)	<0.001

95% CI, 95% confidence interval.

a Adjusted linear regression coefficients

b Multiple Linear Regression (*R*^2^ = 0.601)

The association between the comorbidities of patients with Ps and the high CVR according to the Framingham score and the QIMT was evaluated and is described in table 5 which summarizes the results found, in which we can verify statistically significant associations between the high Framingham score with hypertension, diabetes, triglycerides ≥ 150 mg/dL and cardiovascular disease. However, for high QIMT values, the only statistically significant association was found with LDL ≥ 130 mg/dL.

**Table 5:** Prevalence of comorbidities of patients with psoriasis and high cardiovascular risk, classified by the Framingham score and QIMT.

Comorbidities	High risk Framingham	High risk QIMT
Hypertension	11 (61.1%)^a^	14 (43.8%)
Diabetes	7 (38.9%)^a^	6 (18.8%)
Smoking	5 (27.8%)	7 (21.9%)
Total cholesterol ≥ 200 mg/dL	8 (44.4%)	13 (40.6%)
HDL ≤ 60 mg/dL	15 (83.3%)	25 (78.1%)
LDL ≥ 130 mg/dL	5 (27.8%)	5 (15.6%)^a^
Triglycerides ≥ 150 mg/dL	11 (61.1%)^a^	13 (40.6%)
Cardiovascular disease	6 (33.3%)^a^	6 (18.8%)
High abdominal circumference	16 (88.9%)	28 (87.5%)
Overweight + obesity	13 (72.2%)	28 (87.5%)
Obesity	4 (22.2%)	11 (34.4%)
Metabolic syndrome	12 (66.7%)	17 (53.1%)

a *p* < 0.05 (Chi-square test).

## Discussion

The association between cardiovascular disease and Ps was described almost 50 years ago, but in the last decade there has been an increase in the investigation of this correlation.^10^, ^11^

The inflammatory response in Ps leads to insulin resistance, endothelial dysfunction, oxidative stress and development of atherosclerosis that culminates with Acute Myocardial Infarction (AMI) or stroke. Therefore, the Ps may be considered an independent risk factor for cardiovascular events.^12^

In 2018, Fernandes-Armenteros et al. published an observational study through a database with 398,701 individuals, including 6868 cases of Ps, of which 7.3% were considered moderate to severe Ps, the MS was higher prevalence in patients with Ps (28.3% × 15.1%, OR = 2.21).^13^ Another cross-sectional study containing 244 patients with Ps and another 163 in a control group found a significant prevalence of MS in the Ps group (45.1% vs. 19.6%), regardless of disease severity.^14^ In this study, 50% of patients with MS were found, all with moderate to severe Ps, demonstrating the association of Ps and MS.

A survey of 6549 Americans aged 20–59 years showed that the prevalence of MS (according to the NCEP ATP III criteria reviewed) was 40% among individuals with Ps and 23% among those without Ps, again approaching the data found in this study with 50% of patients with MS. And the most common MS characteristic among the patients was obesity, followed by hypertriglyceridemia and after low levels of HDL cholesterol.^15^

The consequences of comorbidities of Ps, such as higher values of MS, atherosclerosis and CVR, lead to an increase in mortality in these individuals. Gelfand et al. reported that men with severe Ps die 3.5 years earlier than men without Ps and women with severe Ps died 4.4 years earlier than those without Ps. Therefore, modification of the risk factor is necessary to reduce CVR.^1^

Theoretically, the inflammatory events that are present in the blood circulation of individuals with Ps may influence the appearance of vascular endothelial lesions and the development of atherosclerosis. Therefore, the hypothesis that reducing the inflammatory load would consequently decrease the risk of cardiovascular disease.^16^, ^17^ Therefore, the medication chosen for the treatment of Ps should be analyzed not only to improve the skin disease, chronic inflammation and microcirculation, preventing future damage to the individual.

In addition to receiving MTX in Ps therapy as an anti-inflammatory, antiproliferative and immunosuppressive agent, there is considerable evidence of its anti-atherosclerotic effect. A study involving 1240 patients observed over 6 years showed that in both patients with Ps and those with rheumatoid arthritis, MTX reduced the risk of coronary heart disease and also reduced the risk of death from CVD by 70% comparison with patients not treated with MTX.^18^

There is a study evaluating the effects of MTX on the development of atherosclerosis in patients with Ps, which describes its impact on endothelial function in the microcirculation as an early marker of atherosclerosis. After 8–10 weeks of treatment, no significant changes in microcirculation were observed.^19^ As the present cross-sectional study, this limitation occurred to assess whether MTX or TNF-i would reduce CVR after a given time of use medication.

Several recent publications have suggested that TNF-i also has a beneficial impact on CVR.^20^, ^21^, ^22^ In a meta-analysis, Westlake et al. summarized the potential effects of anti-TNF-α in patients with psoriatic arthritis on major adverse cardiovascular events (MACE) and on the risk of developing cardiovascular disease again. The results showed a potential cardioprotective effect of anti-TNF-α, but not as effective as that observed with the use of MTX.^23^

Another study with 2400 patients with severe Ps found that patients treated with MTX or immunobiologicals had low rates of cardiovascular events compared to other therapies.^24^

A systematic review and meta-analysis showed the relationship between the use of TNF-i, MTX, non-steroidal anti-inflammatory drugs and corticosteroids and the presence of cardiovascular events in 236,525 patients with rheumatoid arthritis and 220,209 patients with psoriatic arthritis and Ps. In rheumatoid arthritis, a beneficial association of MTX with cardiovascular risk reduction was found (RR = 0.72, 95% CI 0.57–0.91, *p* = 0.007). However, MTX was not associated with reduced risk of stroke and MACE. The use of TNF-i was associated with the reduction of the CVR (RR = 0.7, 95% CI 0.54–0.9, *p* < 0.005), such as AMI, stroke and MACE. In the analysis of psoriatic arthritis and Ps, the data were sufficient to evaluate the effect of systemic therapy compared with non-use of it or only topical medication. Systemic therapy was associated with decreased CVR (RR = 0.75, 95% CI 0.63–0.91, *p* = 0.003).^25^

Carotid US measuring the medial-intimal thickness are a non-invasive method to evaluate the initial changes of the atherosclerotic vascular wall. When these values are increased, they reflect local abnormalities that correlate with histologically proven atherosclerosis.^26^ Measurement of this thickness has a predictive value in terms of cardiovascular events, regardless of traditional risk factors.^27^

Considering the fact that US is the most operator-dependent imaging technique and US vascularization requires experience and expertise, a method that allows an automated measurement of IMT can facilitate its use, such as automatic high-accuracy and real-time evaluation by radiofrequency, through a software, called QIMT. The manual measurements are influenced by subjective parameters such as the difficulty of the human eye to differentiate the interfaces of the layers and consequently the threshold of the interface of the echo of the eye and the sensitivity of the hand of the sonographer in the positioning of the electronic meters. By the QIMT method there is no such influence on the B-mode image quality, which makes it less dependent on the operator.^7^

In this study subclinical changes consistent with atherosclerosis were seen based on an increased mean QIMT. A previous study with 30 patients with Ps (*n* = 15) and psoriatic arthritis (*n* = 15) demonstrated the relationship between such disorders and an increased risk of subclinical atherosclerosis and cardiovascular events using the QIMT method. Statistically significant values showed that 60% of patients with Ps and 80% with psoriatic arthritis had a greater measure of IMT than expected.^28^

The values found in this study were similar to those published in the literature, in which 64% of the 50 patients with Ps presented altered QIMT levels (56% of MTX users and 72% of individuals receiving TNF-i), while only 36% had intermediate or high risk in the Framingham Score (44% of Group 1 and 28% of Group 2). This shows the usefulness of IMT for alertness of atherosclerosis not yet clinically apparent and in the therapeutic decision initiative of the patient with a chronic inflammatory disease such as Ps. The difference between the QIMT measurement values between the two drug groups (56% of MTX users and 72% of individuals receiving TNF-i) can be attributed to a more important inflammatory process in patients in the group of TNF-i, which had 80% PASI > 10 and only 40% of the MTX group had PASI > 10. Recalling that all patients presented PASI equal to or greater than 10, being evaluated as moderate to severe Ps. In addition, it is known that in clinical practice TNF-i is used for patients with more severe conditions of Ps or therapeutic failures with other medicines, in other words, it is a patient probably with longer disease and greater systemic inflammation, characterizing a more evidenced atherosclerotic process. However, longitudinal studies with a larger number of participants should be performed to confirm these data.

In a systematic review of the literature, twenty-four articles were analyzed (1120 patients and 943 from the control group) to assess the relationship between IMT and ankylosing spondylitis, and treatment with TNF-i suggested an improvement or decrease in process progression of atherosclerosis, whereas inflammatory diseases may induce the development of atherosclerosis.^9^, ^29^

A prospective Spanish study of 53 patients with moderate to severe Ps, also used QIMT, automated radiofrequency method, before and after 8 months of systemic therapy. QIMT of immunobiological users tended to decrease, insulin and glycemia levels decreased with TNF-i and patients undergoing MTX treatment showed a significant decrease in QIMT, concluding that carotid thickness and its consequences may be benefitted from the use of systemic drugs such as biological and MTX.^30^ These results are in agreement with previous studies in patients with rheumatoid arthritis^2^, ^31^ and emphasize that MTX treatment serves as a protective factor during the development of MACEs.^32^

In a pilot study with 16 patients with severe Ps, carotid IMT was calculated by conventional ultrasonographic method before and after 6 months of treatment with TNF-i. A significant decrease in IMT was observed, showing that effective inhibition of tumor necrosis factor decreases IMT in psoriatic patients, and also revealed altered carotid IMT in 84% of patients.^33^

Previously, Di Minno et al. had presented a cross-sectional study comparing IMT levels of patients receiving TNF-i and anti-rheumatic disease modifying drugs for psoriatic arthritis and showed that patients receiving the biological had a decrease in IMT values.^34^

Therefore, it is well documented that IMT of the common carotid artery shows positive correlations with the duration of inflammation, laboratory parameters, age and traditional risk factors for CVD.^35^, ^36^ Cardiology societies suggest that the IMT assessment of the carotid artery should be part of the medical evaluation of patients with increased CVR,^37^ and the guidelines for rheumatology suggest that patients with inflammatory arthritis should undergo ultrasonographic evaluation of the carotid arteries.^38^ However, in the area of dermatology there is no positioning regarding this evaluation imaging, and known to be a chronic inflammatory disease and an independent risk factor for CVD, this subject should be addressed and targeted to the experts in the area as soon as possible.

In the present study, there was a moderate to a strong positive association after assessing the QIMT values correlated with the Framingham score values (*p* < 0.001), showing that QIMT can be considered a possible screening test for cardiovascular risk assessment in patients with Ps. More than 70% of the patients in Group 2 had ITQI altered, while 56% of Group 1 presented alterations, showing lower CVR in patients submitted to MTX treatment. However, when assessed by the Framingham score, Group 1 presented 28% of high CVR and Group 2 20%, noting that the use of ultrasonography by radiofrequency technology should be considered as an instrument for the early detection of atherosclerosis, still without signs clinical or laboratory.

There are some limitations in this study that include the heterogeneity of age and time of use of the medications. While the findings of our study provide supporting evidence, it is important to note that cross-sectional analysis does not provide information on causality and the protective or non-protective effect of the therapies cited in relation to CVR has not been evaluated. Another limiting factor is the small number of participants, due to the cost of biological medications and to follow criteria for the introduction of these medications.

Our study is one of the few to present new ultrasound variables, including an automated measurement of the carotid and middle layer of the carotid artery by radiofrequency and the correlation with other variables in patients with Ps. All ultrasound measurements were performed by a physician with extensive experience in US, and it has been shown that physicians who are not specialists in vascular US can perform reliable assessments of carotid QIMT.^9^

The evaluation of the possible atherogenic damages caused by an inflammatory process such as that resulting from Ps and the identification of subclinical CVD should be made by all professionals in the area and should alert the patient to the risks of CVD and if necessary refer them to the specialist for follow-up and therapy of comorbidities.

The use of US in dermatology is still uncommon, but it is a non-invasive, low-cost and widely accessible method. The techniques are evolving and spectral Doppler ultrasound using the QIMT technique is the most appropriate for inflammatory reactions, allowing the visualization of atherosclerotic changes in the vessel wall caused by the chronic inflammatory activity that occurs in some dermatological diseases, such as Ps.

## Conclusion

There was no statistically significant difference between CVR (measured by Framingham and QIMT) in relation to the treatment two groups (MTX and TNF-i), so it is not possible to state which drug has the highest CVR.

In this study, the association of Ps and MS was verified, as well as very altered results for obesity, waist circumference and BMI.

We also found a moderate to strong positive association correlating the Framingham score values with the QIMT measurement, providing evidence for the use of ultrasound in clinical practice.

## Financial support

None declared.

## Authors’ contributions

Elaine Cristina Faria Abrahão Machado: Approval of the final version of the manuscript; conception and planning of the study; elaboration and writing of the manuscript; obtaining, analysis, and interpretation of the data; effective participation in research orientation; intellectual participation in the propaedeutic and/or therapeutic conduct of the studied cases; critical review of the literature; critical review of the manuscript.

José Alexandre Mendonça: Approval of the final version of the manuscript; conception and planning of the study; obtaining, analysis, and interpretation of the data; effective participation in research orientation; intellectual participation in the propaedeutic and/or therapeutic conduct of the studied cases; critical review of the manuscript.

Ana Carolina Belini Bazán Arruda: Conception and planning of the study; effective participation in research orientation; intellectual participation in the propaedeutic and/or therapeutic conduct of the studied cases.

Luciana Bertoldi Nucci: Statistic analysis conception and planning of the study; obtaining, analysis, and interpretation of the data; critical review of the manuscript.

Marcel Alex Soares dos Santos: elaboration and writing of the manuscript; critical review of the manuscript.

## Conflicts of interest

None declared.
